# Influence of bound dodecanoic acid on the reconstitution of albumin nanoparticles from a lyophilized state

**DOI:** 10.1038/s41598-021-84131-x

**Published:** 2021-02-26

**Authors:** Christian C. E. Luebbert, Rola Mansa, Raisa Rahman, Zygmunt J. Jakubek, Grant E. Frahm, Shan Zou, Michael J. W. Johnston

**Affiliations:** 1 Centre for Biologics Evaluation, Biologics and Genetic Therapies Directorate, Health Canada, 251 Sir Frederick Banting Driveway, Ottawa, ON K1A 0K9 Canada; 2 Metrology Research Centre, National Research Council Canada, 100 Sussex Drive, Ottawa, ON K1A 0R6 Canada; 3 Department of Chemistry, Carleton University, 1125 Colonel By Drive, Ottawa, ON K1S 5B6 Canada

**Keywords:** Nanoscale biophysics, Drug regulation

## Abstract

The development of reference standards for nanoparticle sizing allows for cross laboratory studies and effective transfer of particle sizing methodology. To facilitate this, these reference standards must be stable upon long-term storage. Here, we examine factors that influence the properties of cross-linked albumin nanoparticles, fabricated with an ethanol desolvation method, when reconstituted from a lyophilized state. We demonstrate, with nanoparticle tracking analysis, no significant changes in mean particle diameter upon reconstitution of albumin nanoparticles fabricated with bovine serum albumin loaded with dodecanoic acid, when compared to nanoparticles fabricated with a fatty acid-free BSA. We attribute this stability to the modulation of nanoparticle charge-charge interactions at dodecanoic acid specific binding locations. Furthermore, we demonstrate this in a lyophilized state over six months when stored at − 80 °C. We also show that the reconstitution process is readily transferable between technicians and laboratories and further confirm our finding with dynamic light scattering analysis.

## Introduction

The development of nanoscale drug delivery systems (NDDS) has accelerated over the past decades with several systems approved for clinical use for a wide range of diseases including treatment of cancer and fungal infections^1,2^. Liposomal drug delivery systems have been the most successful delivery systems, including liposomal formulations of doxorubicin (Myocet and Doxil), vincristine (Marqibo), amphotericin B (Abelcet) and Estrogen (Estrasorb)^3,4^. Additionally, a protein nanoparticle formulation of paclitaxel (Abraxane)^5^ and PEGylated interferons^6^ have also obtained regulatory approval. NDDSs can be considered as non-biological complex drugs (NBCD)^7,8^ and one strategy to aid in the regulation of both innovator and follow-on nanomedicines or NBCDs is the appropriate development and use of standards. These standards enable method validation, inter-laboratory comparisons, measurement controls, instrument calibration and performance testing of these products. The need for the development of appropriate reference materials for nanomedicines was further noted by the Global Summit on Regulatory Science in 2016^9^.

We and other researchers have studied cross-linked albumin nanoparticles and have observed these particles to be highly stable and their manufacture to be highly reproducible, in addition to the fact that they are able to deliver therapeutic medicines^10–14^. We proposed these particles as a suitable reference material for particle sizing of protein nanoparticles and submicron protein aggregates. One critical criterion these nanoparticles must meet to serve as a reference material is long-term storage stability, preferably in a lyophilized state. Previous studies have investigated how albumin nanoparticles behave upon freeze drying and reconstitution^15,16^. Anhorn and coworkers observed little change in human serum albumin (HSA) nanoparticle diameter upon reconstitution of empty HSA nanoparticles at lower concentrations (10 mg/mL total protein), but increases in particle diameter (30–50% increase) and polydispersity were observed with increasing protein concentrations (25 mg/mL total protein), depending on the excipient used^15^. Dadpavar and coworkers also observed an approximate 10–35% increase in albumin nanoparticle diameter after reconstitution, again depending on the excipient used^16^.

We have observed that dodecanoic acid has a significant impact on nanoparticle diameter when bound to albumin during the nanoparticle fabrication process^12^, and previous studies have shown that protein-bound fatty acids can improve albumin thermal and chemical stability^17–20^. It was of interest to us to determine how albumin-bound fatty acids may alter the stability of albumin nanoparticles during freeze drying and subsequent reconstitution of albumin nanoparticles. Here, we investigate the influence of dodecanoic acid on albumin nanoparticle stability upon lyophilisation and subsequent reconstitution of bovine serum albumin (BSA) nanoparticles with the aim of developing particles that demonstrate sufficient stability in a lyophilized state to function as a reference material for the size analysis of protein nanoparticles and sub-micron protein aggregates. Lyophilisation allows for the long-term storage of large volumes of materials and its subsequent distribution and is a standard practice in the production of biologics and vaccines.

## Results

### Optimization of defatted albumin nanoparticle (BSA-DF) fabrication

As previous studies have shown that protein concentration can have a dramatic influence on protein nanoparticle diameter^21^, the influence of protein concentration on the mean and mode diameters of nanoparticles fabricated with defatted bovine serum albumin was assessed. Desolvation and crosslinking of a solution of defatted BSA (BSA-DF) at a concentration of 25 mg/mL produced albumin nanoparticles with the smallest diameter and least variability (Supplemental Fig. S1). As such, all future nanoparticles fabrications were carried out with a protein concentration of 25 mg/mL. The effect of starting protein solution volume was assessed, with volumes ranging (2–25) mL, and it was observed that nanoparticle diameter was essentially volume independent (Supplemental Fig. S1). All further studies were carried with an initial protein solution volume of 10 mL.

### Influence of BSA-DF nanoparticle concentration on freeze drying

**Figure 1:**
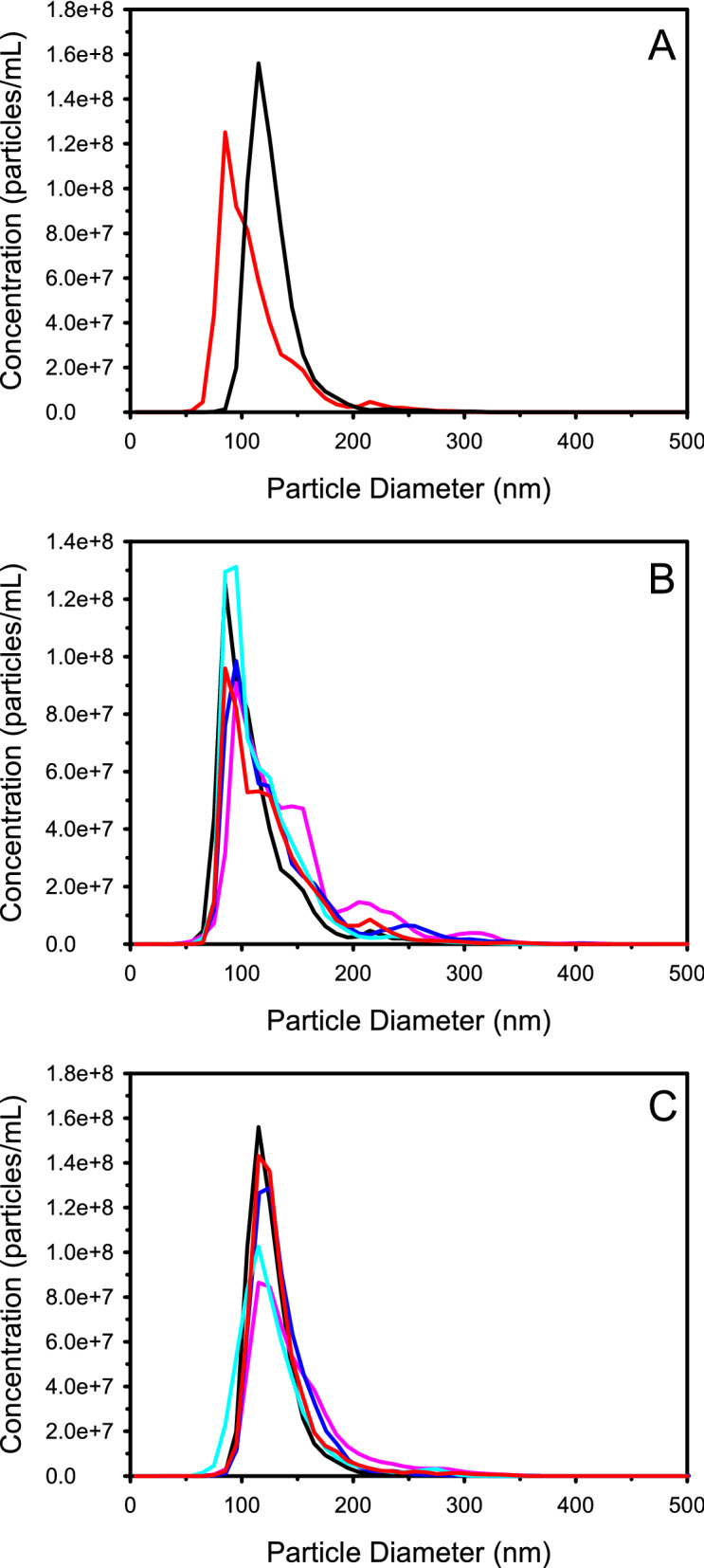
(**A**) Distribution profiles of BSA-DF (red) and BSA-DF-C12 (black) nanoparticle before lyophilization. (**B**) Distribution profiles after reconstitution from a lyophilized state for BSA-DF (black), BSA-DF 33 (pink), BSA-DF 50 (blue), BSA-DF 100 (cyan) and BSA-DF 200 (red). (**C**) Distribution profiles after reconstitution from a lyophilized state for BSA-DF-C12 (black), BSA-DF-C12 10 (pink), BSA-DF-C12 33 (blue), BSA-DF-C12 50 (cyan) and BSA-DF-C12 100 (red). Plots represent the mean from three measurements with nanoparticle tracking analysis.

**Figure 2:**
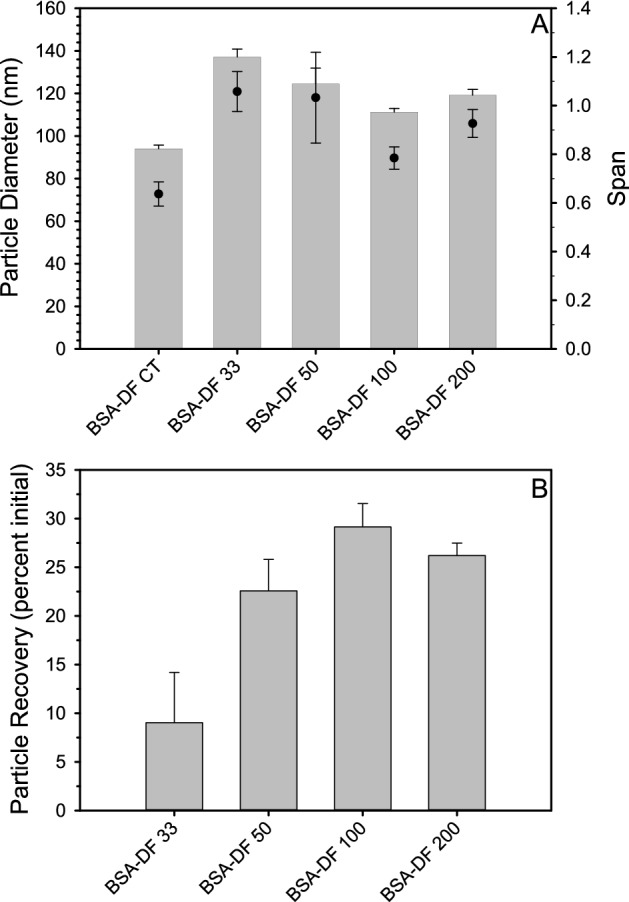
(**A**) Plots of mean diameter (grey bars) and Span (black circles) pre freeze-drying (BSA-DF) and after reconstitution at various particle concentrations for BSA nanoparticles fabricated with defatted protein. (**B**) Plot of particle recovery for freeze-dried BSA-DF nanoparticles at different concentrations upon reconstitution. Data represents mean values from three measurements and error bars represent the standard deviation.

Previous studies have shown that pre-freeze dried nanoparticle concentration can have a dramatic influence on the particle size and polydispersity of reconstituted nanoparticles^15^. Non-lyophilized BSA-DF (BSA-DF CT) nanoparticles were analyzed with nanoparticle tracking analysis (NTA) to determine particle concentrations, and aliquots were subsequently lyophilized at 200, 100, 50 and 33 percent of the initial non-lyophilized nanoparticle concentration (BSA-DF 200, 100, 50 and 33: 1.08 × 10^13^, 5.04 × 10^12^, 2.5 × 10^12^ and 1.68 × 10^12^ particles/mL, respectively). Plots of the particle profiles showed that pre-freeze dried BSA-DF (Fig. 1A) was asymmetrical with a few large aggregates, and that the increased presence of aggregates at 200 and 300 nm was noted with reconstituted BSA-DF nanoparticles (Fig. 1B). Reconstitution of freeze-dried BSA-DF nanoparticles at all concentrations resulted in increases in mean diameters as well as an increase in particle population span, with reconstitution at 100% of the initial fabrication concentration (no dilution from the concentration as fabricated) showing the smallest increases (Fig. 2A). Recovery of BSA-DF nanoparticles ranged from approximately 10 to 30% post-reconstitution, with reconstituted aliquots of BSA-DF 100 showing the greatest particle recovery (Fig. 2B).

### Influence of BSA-DF-C12 nanoparticle concentration on freeze drying

Nanoparticles fabricated with fatted BSA (dodecanoic acid) (BSA-DF-C12) at a protein concentration of 25 mg/mL demonstrated the lowest variability and smallest difference in mean diameters (Supplemental Fig. S2) and all further fabrications of BSA-DF-C12 nanoparticles were carried out at that protein concentration.

**Figure 3:**
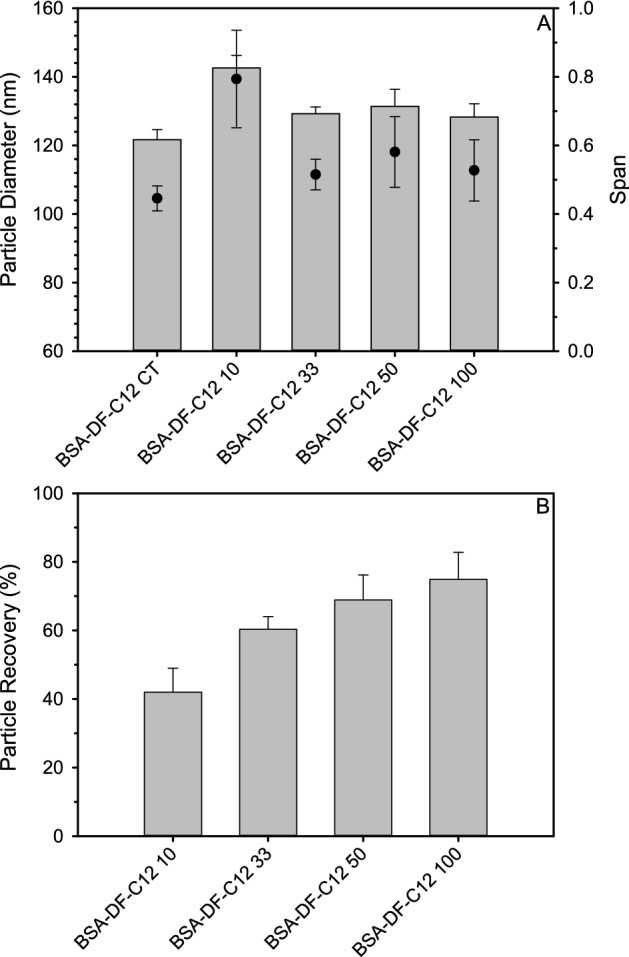
(**A**) Plots of mean diameter (grey bars) and Span (black circles) pre freeze-drying (BSA-DF-C12) and after reconstitution at various particle concentrations for BSA nanoparticles fabricated with dodecanoic acid loaded protein. (**B**) Plot of particle recovery for freeze-dried BSA-DF-C12 nanoparticles at different concentrations upon reconstitution. Data represents mean values from three measurements and error bars represent the standard deviation.

Nanoparticle tracking analysis resulted in distribution plots of BSA-DF-C12 nanoparticles which were more symmetrical than those for BSA-DF nanoparticles with no observable aggregates (Fig. 1A). BSA-DF-C12 nanoparticles were lyophilized at 100% of the fabricated concentration, as well as at 50%, 33%, and 10% dilutions (BSA-DF-C12 100, 50, 33 and 10: 1.57 × 10^13^, 7.8 × 10^12^, 5.2 × 10^12^ and 1.57 × 10^12^ particles/mL, respectively), matching the particle concentration ranges of BSA-DF studies. Unlike reconstituted BSA-DF nanoparticles, no aggregates were observed upon reconstitution, although a broadening of the peak shoulder for BSA-DF-C12 10 and 33 were noted (Fig. 1C). Similar mean diameter and span values were observed for BSA-DF-C12 100, 50 and 33, while BSA-DF-C12 10 showed a dramatic increase in both mean particle diameter and particle population span (Fig. 3A).

The efficiency of the nanoparticle reconstitution was evaluated by calculating the number of particles that were recovered and it was determined that for BSA-DF-C12 100, 50 and 33 particle counts remained between approximately 60% and 80% upon reconstitution (Fig. 3B). BSA-DF-C12 10 demonstrated only 42% particle recovery. All further lyophilisation studies were carried out with nanoparticle concentrations undiluted from their initial fabrication concentration (BSA-DF-C12-100).

### Assessment of freeze-drying for multiple fabrication lots of BSA-DF and BSA-DF-C12 nanoparticles

Multiple lots (3) of BSA-DF nanoparticles were produced. At least three aliquots from each fabrication were assessed with NTA prior to freeze-drying and at least three aliquots were freeze-dried, reconstituted and assessed with NTA. BSA-DF production appeared highly reproducible with coefficients of variation below 5% for mean diameters for the three fabrication lots produced. However, significant changes were seen in the average mean particle diameter after reconstitution (P ≤ 0.001) with an average increase of 16 nm, representing an approximate seventeen percent increase in nanoparticle size (Table 1). Significant changes were also observed with the span (P ≤ 0.001) values when compared with pre-lyophilized BSA-DF particles (Table 1).

**Table 1:** Nanoparticle tracking assessment of BSA-DF and freeze dried/reconstituted BSA-DF nanoparticles from multiple fabrication lots (3).

	Mean diameter (nm)	D10 (nm)	D50 (nm)	D90 (nm)	Span
**Pre-lyophilized BSA-DF nanoparticles**					
Mean ± SD	93 ± 1	71 ± 1	84 ± 2	108 ± 1	0.44 ± 0.03
COV (%)	1.1	1.4	2.4	1.0	6.8
**Reconstituted BSA-DF nanoparticles**					
Mean ± SD	109 ± 1	81 ± 4	94 ± 2	143 ± 5	0.66 ± 0.03
COV (%)	1.0	4.8	2.1	3.5	4.5

*SD* standard deviation of the mean.

Fabrication of multiple lots (5) of BSA-DF-C12 nanoparticles was undertaken. At least three aliquots from each fabrication were assessed with NTA prior to freeze-drying and at least three aliquots were freeze-dried, reconstituted and assessed with NTA. Analysis of pre-freeze dried BSA-DF-C12 nanoparticles showed that fabrication was highly reproducible, with variation in mean and mode values of less than 5% over the time period of nanoparticle fabrication. Freeze-dried and reconstitution of BSA-DF-C12 nanoparticles did not show significant changes in either mean (P = 0.188) or Span values (P = 0.149) (Table 2).

**Table 2:** Nanoparticle tracking assessment of BSA-DF-C12 and freeze dried/reconstituted BSA-DF-C12 nanoparticles from multiple fabrication lots (5).

	Mean Diameter (nm)	D10 (nm)	D50 (nm)	D90 (nm)	Span
**Pre-lyophilized BSA-DF-C12 nanoparticles**					
Mean ± SD	120 ± 4	93 ± 2	111 ± 4	141 ± 6	0.43 ± 0.02
COV (%)	3.3	2.2	3.6	4.3	4.7
**Reconstituted BSA-DF-C12 nanoparticles**					
Mean ± SD	124 ± 4	94 ± 3	113 ± 4	149 ± 8	0.48 ± 0.03
COV (%)	3.2	3.2	3.5	5.4	6.3

*SD* standard deviation of the mean.

### Assessment on the transferability for the reconstitution of lyophilized BSA-DF-C12 nanoparticles

To assess the protocol’s transferability, a second technician was instructed on the reconstitution method. Retained pre-lyophilized and lyophilized samples of BSA-DF-C12 nanoparticles from four rounds of albumin nanoparticle fabrication previously reconstituted and assessed by Technician #1 were reconstituted and assessed by Technician #2 on the NS300 instrument (Table 3). Comparison of results between Technician #1 and Technician #2 shows no significant differences in mean diameter (P = 0.596) or Span (P = 0.371) values of the reconstituted BSA-DF-C12 lots assessed with the coefficient of variation for each technician being similar. This would suggest that the reconstitution protocol is readily transferable.

**Table 3:** Comparison of the reconstitution of BSA-DF-C12 between technicians.

Fabrication lot#	Mean diameter (nm)	D10 (nm)	D50 (nm)	D90 (nm)	Span
**Reconstituted BSA-DF-C12 nanoparticles—technician #1**					
051018	127	98	118	151	0.45
181018	124	96	115	146	0.43
301118	119	92	109	141	0.46
071218	120	92	108	146	0.51
Mean ± SD	123 ± 4	95 ± 3	113 ± 4	146 ± 4	0.46 ± 0.03
COV (%)	3.3	3.2	3.5	2.7	6.5
**Reconstituted BSA-DF-C12 nanoparticles—technician #2**					
051018	125	96	115	150	0.47
181018	120	93	111	141	0.43
301118	120	92	109	150	0.53
071218	120	91	107	149	0.54
Mean ± SD	121 ± 3	93 ± 2	111 ± 3	148 ± 4	0.49 ± 0.05
COV (%)	2.5	2.2	2.7	2.7	10.2

*SD* standard deviation of the mean.

### Assessment of storage of lyophilized BSA-DF-C12 nanoparticles over 6 months

Stability during long-term storage is a desirable trait for reference materials. To assess the long-term storage of nanoparticles, multiple vials from one fabrication lot of BSA-DF and BSA-DF-C12 were lyophilized and stored at − 80 °C for 6 months. The retained vials were reconstituted, and analysis showed that BSA-DF-C12 nanoparticles are stable over an extended period (Table 4). No difference in the reconstitution process for nanoparticles at T = 0 or T = 6 months was observed.

**Table 4:** Nanoparticle tracking assessment of reconstituted BSA-DF and BSA-DF-C12 nanoparticles 6 months post-lyophilisation.

Mean diameter (nm)	D10 (nm)	D50 (nm)	D90 (nm)	Span
**Reconstituted BSA-DF-C12 nanoparticles T = 0 Lot# 181018**				
124 ± 2	96 ± 2	115 ± 2	146 ± 6	0.43
**Reconstituted BSA-DF-C12 nanoparticles T = 6 months**				
120 ± 2	94 ± 1	111 ± 1	139 ± 2	0.41

Data represents mean values ± standard deviation from measurement of at least three aliquots of nanoparticles.

### Assessment of lyophilized albumin nanoparticles with a second nanoparticle tracking instrument and dynamic light scattering

To ensure our observations were not instrument specific, Technician #1 assessed reconstituted BSA-DF-C12 nanoparticles on a Nanosight NS500 instrument in a different facility. Similar results were observed as with the NS300 instrument, with BSA-DF-C12 remaining stable post-lyophilisation (Supplemental Table S1). We also assessed reconstituted BSA-DF-C12 nanoparticle with dynamic light scattering (DLS), a commonly used technique for the assessment of nanoparticles and nanomedicines^22^. Five lyophilized vials of BSA-DF-C12 nanoparticles were reconstituted and diluted to appropriate concentrations. Our analysis showed that reconstituted BSA-DF-C12 nanoparticles were monomodal and the presence of agglomerates were not observed (Supplemental Table S2). Mean Z-average (141 nm ± 5 nm) and mean distribution (154 nm ± 7 nm) diameters showed low variability between multiple reconstituted vials. Additionally, a mean polydispersity index (0.08 ± 0.03) was noted, further indicating the monodisperse nature of reconstituted BSA-DF-C12 nanoparticles. It was also seen that particle diameters were larger than those reported with nanoparticle tracking analysis with the mean distribution diameter values largest and the NTA diameter values smallest of the three. The trend of diameter values was as expected^22–24^ since the definitions of the diameter measured were different with the Z-average diameter being an intensity weighted harmonic average, the mean distribution diameter being an intensity weighted arithmetic average, and the NTA diameter being a number weighted arithmetic average. While a conversion between intensity and number weighted distributions is not straightforward and generally should not be attempted, it can be shown that the two DLS-determined mean diameter values are consistent with the corresponding NTA mean values.

Zeta potential values for the prelyophilized and reconstituted BSA-DF and BSA-DF-C12 nanoparticles was also assessed (Supplemental Table S3) with zeta potential values for lyophilized samples appearing more negative. This suggests some structural changes to the protein nanoparticles during lyophilisation and subsequent reconstitution.

### Assessment of reconstituted BSA-DF C12 nanoparticles with Atomic Force Microscopy

**Figure 4:**
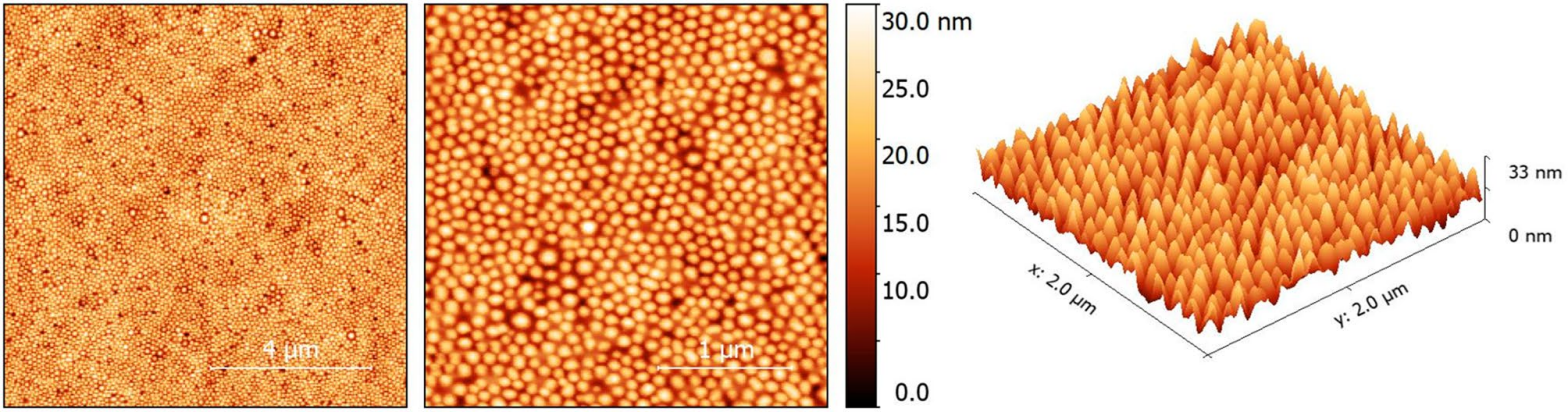
Atomic force microscopy images of BSA-DF-C12 lyophilized sample spin-coated and measured in ambient.

Reconstituted BSA-DF C-12 nanoparticles were assessed with atomic force microscopy (AFM) and showed these nanoparticles are highly uniform in size when lyophilized (Fig. 4), in agreement with our previous scanning electron microscopy evaluation of pre-lyophilized BSA-DF and BSA-DF C12 nanoparticles^12^.

### Assessment of additional anions on reconstituted lyophilized BSA-DF nanoparticles

We hypothesized/theorized that the lack of changes in particle parameters upon reconstitution of BSA-DF-C12 nanoparticles (relative to BSA-DF nanoparticles) was due to the close association of the carboxyl end of the C12 fatty acid with positively charged residues in fatty acid binding sites, and to the associated changes in nanoparticle-nanoparticle electrostatic interactions during freeze-drying. To investigate this, BSA-DF nanoparticles were fabricated with excess carboxyl ions. This was done by lowering the initial pH of BSA-DF solution (prepared at pH 8.5) to pH 7.8 with methanoic (formic) acid (FA) (this matches the downward pH shift when dodecanoic acid and BSA-DF are incubated at a 6:1 molar ratio). The BSA-DF solution was then readjusted to pH 8.5 prior to desolvation. Reconstitution of these freeze-dried nanoparticles (BSA-DF-FA) resulted in little or no change in mean diameter or span values (Supplemental Table S4).

### Assessment of nanoparticle diameter on the behaviour of albumin nanoparticles reconstituted from a lyophilized state

To determine if the behaviour upon reconstitution from a lyophilized state for BSA-DF-C12 and BSA-DF-FA nanoparticles is specific to compounds containing carboxyl groups or can be brought about by other ions, we fabricated nanoparticles with excess chloride ions. This was done by adjusting the pH of the protein solution prior to desolvation with HCl as described above and previously^12^. Freeze-drying and reconstitution of these nanoparticles (BSA-DF-Cl) showed increases in mean particle diameters and particle span values similar to BSA-DF nanoparticles (Supplemental Table S4).

Furthermore, nanoparticles were fabricated with a protein solution with a pH of 7.5 (solubilized and pH adjusted to 7.5 with NaOH, with no further adjustment) to determine if the increased diameter of BSA-DF-C12 nanoparticles, compared to BSA-DF nanoparticles, is responsible for their observed performance upon reconstitution. These larger BSA-DF-7.5 nanoparticles were similar in diameter to BSA-DF-C12 nanoparticles but upon reconstitution from a lyophilized state demonstrate increases in mean diameters and span values similar to BSA-DF and BSA-DF-Cl nanoparticles. This suggests that particle size is not responsible for the insignificant changes in the mean diameter of BSA-DF-C12 nanoparticles upon reconstitution from a lyophilized state (Supplemental Table S5).

## Discussion

The availability of nanoparticle reference materials enables the fast development and improved regulation of nanomedicines and other nanotechnology enabled products. We proposed the development of bovine serum albumin nanoparticles fabricated with a well-established desolvation and crosslinking method as a suitable reference material for particle sizing of protein nanoparticles and submicron protein aggregates. This study demonstrates that the production of nanoparticles composed of fatted BSA (BSA-DF-C12) is highly reproducible and can be readily freeze-dried and stored for an extended period with no significant changes in mean nanoparticle diameter when reconstituted from a lyophilized state. This investigation also demonstrates this behaviour is observable with nanoparticle tracking analysis, as well as dynamic light scattering and the methodology for the reconstitution of freeze-dried nanoparticles is readily transferable between technologists.

Nanoparticles composed of fatted albumin (BSA-DF-C12) offer superior physical stability (maintaining particle diameter upon reconstitution) compared to nanoparticles fabricated with defatted albumin (BSA-DF) when reconstituted from a lyophilized state. We observed the generation of aggregates and corresponding significant changes to BSA-DF mean diameter and particle population span upon particle reconstitution. These changes were not observed with reconstituted BSA-DF-C12 nanoparticles. Additionally, we noted greater changes with zeta potential values for reconstituted BSA-DF nanoparticles in comparison to BSA-DF-C12 nanoparticles.

There are a number of mechanisms by which this could occur. The first is a concentration-dependent phenomenon, as the reconstitution of the lowest concentrations of freeze-dried BSA-DF and BSA-DF-C12 nanoparticles showed the greatest changes to particle distribution profiles. This contrasts with previous studies which have shown that nanoparticles at higher concentrations will aggregate more readily for a constant concentration of excipient^15^, although studies here use a much higher concentration of cryoprotectant. Furthermore, the effect of protein concentration on aggregation remains a complex issue^25^, with studies showing increasing protein concentration either enhancing or reducing th*e* propensity for protein to aggregate^26^, or shifting the size of aggregates^27^. It remains unknown how these observations would translate to the aggregation of protein nanoparticles. The studies presented here suggest that concentration, at the working concentrations used (BSA-DF-100 or BSA-DF-C12-100), does not contribute to the improved stability of BSA-DF-C12 over BSA-DF nanoparticles. Increasing the concentration of BSA-DF nanoparticles during the freeze drying process (i.e.-BSA-DF-200—1.08 × 10^13^ particles/mL) to that of BSA-DF-C12-100/50 ((1.57 × 10^13^ to 7.8 × 10^12^) particles/mL), or vice-versa, did not result in BSA-DF demonstrating the stability or particle recovery of BSA-DF-C12, or in BSA-DF-C12 demonstrating the increase in span and polydispersity (and loss of particle recovery) of BSA-DF upon reconstitution.

A second possibility is BSA-DF-C12 nanoparticles are larger than BSA-DF nanoparticles resulting in an approximate 20% decrease in surface to volume ratio of BSA-DF C12 compared to BSA-DF for a given number of nanoparticles. This could result in decreased particle–particle interaction and, ultimately, less particle aggregation for BSA-DF-C12 nanoparticles. However, when nanoparticles with similar size (BSA-DF-7.5 or BSA-DF-Cl nanoparticles) were freeze-dried and reconstituted, they behaved in a similar manner as BSA-DF nanoparticles, with large changes in mean diameters and span values. This would suggest that particle size is not playing a significant role in the improved stability of BSA-DF-C12 over BSA-DF nanoparticles.

A third possible explanation is founded on multiple studies that show protein bound fatty acids enhance albumin structural stability when challenged with thermal or chemical stress^18–20^. Studies investigating albumin behavior during lyophilisation have shown changes to protein secondary structure during the lyophilisation process, with this structural disruption associated with aggregation via thiol-disulfide interchange^28,29^. Changes in the structure of individual proteins that compose BSA-DF nanoparticles could allow for aggregation of the nanoparticles through thiol-disulfide interchange or interactions of previously buried domains. Conversely, protein bound dodecanoic acid may enable the individual proteins composing BSA-DF-C12 nanoparticles to retain more of their native structure during either the fabrication or the lyophilisation process thereby reducing nanoparticle-nanoparticle interaction during reconstitution. This explanation is supported by the Zeta potential measurement in which the change for reconstituted BSA-DF-C12 nanoparticles was dramatically less than that of BSA-DF nanoparticles suggesting less change to the surface composition the fatted nanoparticles.

Finally, bound fatty acids may alter nanoparticle electrostatic interactions during lyophilisation and reconstitution through the close association of the carboxyl end of the fatty acid with positively charged residues in fatty acid binding sites on the protein^30^. We observed no changes in measured parameters of measured parameters (mean particle diameter, D10, D50 and D90 values) for BSA nanoparticles fabricated with dodecanoic acid (BSA-DF-C12) or formic acid (BSA-DF-FA) but not chloride ions (BSA-DF-Cl). The lack of change in particle diameter afforded by the presence of carboxylic anions but not chloride anions would suggest a degree of site specificity and alterations in localized protein–protein interactions. We previously observed a similar phenomenon when assessing the impact of bound fatty acids on the fabrication of albumin nanoparticles, where albumin-bound dodecanoic acid resulted in increased nanoparticle diameter^12^.

In conclusion, we assessed the stability of BSA-DF and BSA-DF-C12 nanoparticles after reconstitution from a lyophilized state and demonstrated significant changes in mean particle diameter and particle population span, the generation of aggregates and greater changes to the zeta potential for of BSA-DF nanoparticles compared to BSA-DF-C12 nanoparticles. Taken in totality, our results suggest that these observations are due to improved structural stability and/or moderation of particle/particle interactions afforded by the presence of the fatty acids. Furthermore, we believe that BSA-DF-C12 nanoparticles could serve as a basis for the development of reference materials for NTA assessment of protein-based nanoparticles and submicron protein aggregates.

We believe that protein nanoparticle reference materials would be particularly useful for NTA assessment of submicron protein aggregates in biologic therapeutics^22,31,32^, which have been associated with adverse events and reduced efficacy. A disadvantage of NTA systems is the analysis being highly dependent on user-defined hardware and software settings^33,34^, potentially complicating the comparison of data or the transfer of methodologies between laboratories. Currently, we and other researchers use polystyrene beads (PS) to verify the NTA instrument is functioning correctly. These particles have properties distinct from protein nanoparticles or protein aggregates, most notably their refractive index (PS beads RI ca 1.59–1.6 and protein particles RI ca 1.34–1.4) as well as their density (PS beads 1.05 g/cm^3^, protein nanoparticles (1.28–1.33) g/cm^3^^35^). In our experience, using instrument settings optimized for the characterization of PS beads leads to some smaller protein particles being too faint for NTA instrument detection, skewing the reported particle distribution and concentration. To correct this, instrument settings must be optimized using the sample being assessed. The availability of nanoparticle reference materials with similar optical properties to protein-based nanoparticles would ameliorate this. The instrument could be calibrated with material similar to the samples being assessed and this would further ease the comparison of data and transfer of methods between research laboratories^9^. The need for optically similar reference materials has been further noted for the sizing of microvesicles with flow cytometry^36^ and characterization of extracellular vesicles with optical methodologies such as dynamic light scattering (DLS) and flow cytometry^37^. As the variety of materials of research interest grows (as well as the methods and instrumentation used to analyse them), so too should the variety of reference standards available for their characterization.

## Materials and methods

### Materials

Fatty acid-free bovine serum albumin (BSA-DF, ≥ 99% purity, P/N# A0281-5G, Lot# 021M7403) was purchased from Sigma-Aldrich (St. Louis, MO, USA). Amicon Ultra 4.0 mL 3000 Da molecular weight cut-off (MWCO) centrifugal filters were obtained from Millipore (Millipore (Canada) Ltd, Etobicoke, ON, Canada)^12^. All other chemicals were obtained from Sigma-Aldrich.

### Albumin sample preparation

Defatted bovine serum albumin (BSA-DF) was prepared as described previously (12). BSA shows high homology with human serum albumin and was utilized in these studies due to cost and the ready availability of defatted material. Briefly, samples were buffer exchanged into 10 mM NaCl, with Amicon Ultra 4.0 mL 3000 Da MWCO centrifugal filter devices pre-washed with buffer. Protein concentrations were measured using a BCA protein assay kit (Sigma-Aldrich) and with a UV/visible spectrophotometer (Ultrospec 3100 pro, Biochrom Ltd., Cambridge, UK).

### Nanoparticle fabrication

A slightly modified nanoparticle fabrication methodology from previously described was utilized (12) (Supplemental Fig. S3). Each round of nanoparticle fabrication began with freshly solubilized BSA-DF diluted to appropriate protein concentrations and volumes in 10 mM NaCl. Prior studies have identified the interference of buffers in the desolvation process; thus our experiments utilized 10 mM NaCl as an ionic background for the adjustment with NaOH to achieve a final pH of 8.5^11,12,14,21^. To this sample, ethanol was added at a flow rate of 10 mL/min using a digital PHD ULTRA Syringe Pump (Harvard Apparatus, Holliston, MA, USA) while the sample was stirred at 550 rpm. A glutaraldehyde solution (8% in water vol:vol) was then added and the sample was stirred for a further 22 h. The nanoparticles were then centrifuged at 16,100 relative centrifugal force (RCF) for 10 min at 4 °C with the pellet resuspended with ultrapure water (18 MΩ cm, 0.2 μm filtered) followed by a 5-min bath sonication (Branson Ultrasonics, model CPX3800H, Danbury, Connecticut, USA). This washing procedure was repeated two more times prior to a final resuspension in ultrapure water and storage at 4 °C. For preparations assessing the effect of fatty acids on nanoparticle properties, BSA-DF was incubated with fatty acids at 37 °C for 2 h while stirring, followed by a pH readjustment to pH 8.5 with NaOH prior to desolvation.

### Nanoparticle freeze drying

Cross-linked albumin nanoparticles were pelleted (16100 RCF) and resuspended in 50 nm-filtered (GE Healthcare, Mississauga, ON, Canada, cat # 110603) lyophilisation buffer (300 mM sucrose, PBS, pH 7.4). The nanoparticle solution was partitioned into 1 mL aliquots (each fabrication produced 12 × 1 mL aliquots) that were then rapidly frozen in liquid nitrogen (77 K) and then dried under vacuum for 24 h using a FreeZone 2.5 L Benchtop Freeze Dry System (Labconco, Kansas City, MO, USA) at − 40 °C and subsequently stored at − 80 °C.

### Nanoparticle reconstitution

Prior to measurement, freeze dried samples were reconstituted in ultrapure water (18 MΩ cm, 0.2 μm filtered) and vortexed for 1 min, sonicated for 5 min with a bath sonicator, vortexed for 30 s and then treated as non-lyophilized samples.

### Nanoparticle tracking analysis

A NanoSight NS300 or a NanoSight NS500 (Malvern Instruments Ltd, Worcestershire, UK) was utilized for particle sizing and counting of albumin nanoparticles according to the manufacturer’s instructions. Camera levels and sample concentration (using ultrapure water) were adjusted to yield 25–35 particles per frame during capture. Optimum camera levels were determined to be fourteen and thirteen for BSA-DF and BSA-DF-C12 nanoparticles, respectively. Results are reported as average mean diameters, mode, *D10*, *D50* and *D90* values from duplicate measurements of pre-lyophilized nanoparticle samples and from the discrete analysis of at least three vials of reconstituted nanoparticles. Distribution widths were expressed as:

$$Span= \frac{D90-D10}{D50}$$

where 90% of the distribution lies below the *D90* and 10% of the population lies below the *D10*. The D*50* is the median.

### Dynamic light scattering analysis

A Zetasizer Nano ZS (red) (ZEN3600) (Malvern Instruments Ltd, Worcestershire, UK) was utilized to confirm the behaviour of lyophilized albumin nanoparticles upon reconstitution. Samples were diluted in water at 25 °C according to the manufacturer’s instructions. Several intensity weighted size measurements such as the Z-average diameter, polydispersity index, and size distribution were reported.

### Atomic force microscopy

For the BSA-DF-C12 lyophilized sample used in AFM imaging, the nanoparticle solution was vortex-mixed for 15 s. A 25 μL aliquot of BSA-DF-C12 was added onto the freshly cleaved highly oriented pyrolytic graphite (HOPG) substrate (GRADE ZYB 12 × 12 × 2 mm, SPI Supplies, West Chester, PA, USA), which was then vacuum mounted onto a spin coater (WS-650SZ-6NPP/LITE, Laurel, North Wales, PA, USA). The spin coating was performed immediately using static mode at 2000 rpm for 60 s, with an acceleration rate of 100 rpm/s, followed by 4000 rpm for 30 s. The samples were dried under nitrogen stream for 10–20 s.

BSA-DF-C12 samples were imaged using a MultiMode AFM with a NanoScope V controller (Bruker Nano Surfaces Division, Santa Barbara, CA, USA), in PeakForce QNM mode. The use of PeakForce imaging facilitates stable and reproducible use of low (down to 10 pN) imaging force. Silicon nitride ScanAsyst-Air AFM probes (Bruker AFM Probes, Camarillo, CA) were used in all PeakForce imaging. The manufacturer specified nominal tip radius and spring constants are 2 nm and 0.4 N/m, respectively. A series of 10 μm × 10 μm, 4 μm × 4 μm and 2.5 μm × 2.5 μm images were acquired with a resolution of 512 pixels × 512 pixels at a scan rate of 0.8–1.0 Hz.

All AFM images were processed in Gwddyion 2.53 with align rows-median to remove skipping lines first. Then, the images were flattened with the first order leveling excluding masked nanoparticles.

### Statistical analysis

Significance was assessed using the Student’s Paired t-test or the Mann–Whitney Rank Sum Test if the assumptions of the t-test are not met. SigmaPlot 12.5 software (Systat Software, Inc., San Jose, CA, USA) was used and significance was designated as P < 0.05^12^.

## Supplementary Information

Supplementary Information.
