# Supplementary material for: Influence of bound dodecanoic acid on the reconstitution of albumin nanoparticles from a lyophilized state
Source: Sci Rep. 2021 Feb 26;11:4768. doi: 10.1038/s41598-021-84131-x (PMC7910568; doi:10.1038/s41598-021-84131-x)
Supplement: Supplementary file 1 — Supplementary Information. [file 41598_2021_84131_MOESM1_ESM.pdf]

Supplementary Table and Figure for:

Influence of bound dodecanoic acid on the reconstitution of albumin nanoparticles from a lyophilized state

Christian C.E. Luebbert, Rola Mansa, Raisa Rahman, Zygmunt J. Jakubek, Grant E. Frahm, Shan Zou and Michael J.W. Johnston

Supplemental Table S1. Nanoparticle tracking assessment of reconstituted BSA-DF-C12 nanoparticles with NS300 and NS500 nanoparticle tracking analysis

	Mean diameter (nm)	D10 (nm)	D50 (nm)	D90 (nm)	Span
**Reconstituted BSA-DF-C12 Fabrication Lot# 101818**					
**NS300 Instrument**	124 ± 2	96 ± 2	115 ± 2	146 ± 6	0.43
**NS500 Instrument**	123 ± 1	95 ± 2	112 ± 1	142 ± 4	0.41
**Reconstituted BSA-DF-C12 Fabrication Lot# 120718**					
**NS300 Instrument**	120 ± 1	92 ± 1	108 ± 1	146 ± 6	0.51
**NS500 Instrument**	122 ± 1	93 ± 1	109 ± 1	146 ± 1	0.50

Data represents mean values ± standard deviation from at least 3 aliquots.

Supplemental Table S2. Assessment of BSA-DF-C12 nanoparticles with dynamic light scattering (DLS) of five reconstituted aliquots from a single fabrication lot

**Fabrication Lot # 240719**	**Mean Values**	**COV%**
**Z-average diameter (nm)**	141 ± 5	3.5
**Mean distribution diameter (nm)**	154 ± 7	4.5

Supplemental Table S3, Zeta potential measurement of prelyophilized and reconstituted BSA-DF and BSA-DF-C12 nanoparticles

**Sample**	Zeta Potential
BSADF, non-lyophilized	-10.9 ± 3.5
BSADF, lyophilized	-33.6 ± 6.5
BSADF + C12, non-lyophilized	-11.4 ± 0.7
BSADF + C12, lyophilized	-19.3 ± 0.7

Data represents mean values ± standard deviation from at least three measurements of a single fabrication lot

Supplemental Table S4. Assessment of reconstitution of lyophilized albumin particles fabricated with excess carboxyl ions (BSA-DF-FA)

	**Mean Diameter (nm)**	**D10 (nm)**	**D50 (nm)**	**D90 (nm)**	**Span**
**Pre-lyophilisation**	141 ± 1	109 ± 1	133 ± 1	165 ± 4	0.42
**Reconstituted**	140 ± 2	110 ± 1	133 ± 2	162 ± 2	0.39

Data represent mean values, two aliquots were analyzed for pre-freeze-dried samples and three aliquots were measured for reconstituted samples.

Supplemental Table S5. Nanoparticle tracking assessment of reconstituted BSA-DF nanoparticles fabricated at pH 7.5 lyophilized with excess chloride ions

	**Mean Diameter (nm)**	**D10 (nm)**	**D50 (nm)**	**D90 (nm)**	**Span**
**BSA-DF-Cl (nanoparticle fabricated with excess chloride ions)**					
**Pre-lyophilisation**	121 ± 3	94 ± 2	113 ± 2	138 ± 1	0.39
**Reconstituted**	128 ± 2	97 ± 1	118 ± 2	152 ± 7	0.47
**BSA-DF-7.5 (nanoparticle fabricated at pH 7.5)**					
**Pre-lyophilisation**	110 ± 3	83 ± 2	98 ± 2	140 ± 4	0.58
**Reconstituted**	120 ± 1	87 ± 1	105 ± 1	156 ± 5	0.66

Data represent mean values ± standard deviation. Two aliquots of nanoparticles were analyzed for pre-lyophilized and three aliquots for nanoparticles fabricated with excess chloride ions. 4 aliquots each for pre-lyophilized and reconstituted samples for nanoparticles fabricated at pH 7.5.

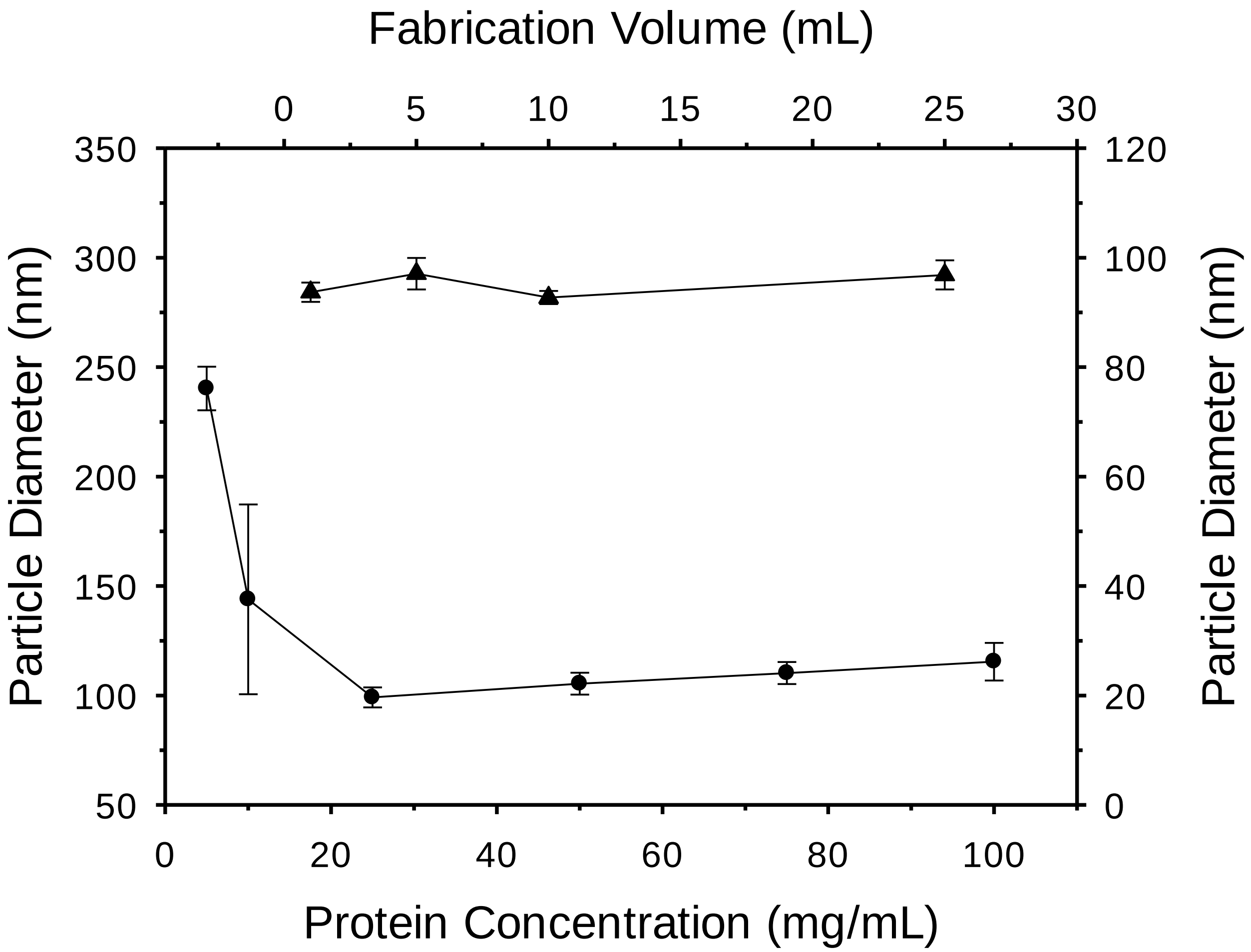

Supplemental Figure S1. Bottom and left axis: effect of initial defatted bovine serum albumin (BSA-DF) concentration on the mean diameter of cross-linked BSA nanoparticles measured with nanoparticle tracking analysis (black circles). Top and right axes: effect of initial protein solution volume on mean nanoparticle diameter (black triangles). Data represents the mean of three measurements from a single fabrication with error bars representing the standard deviation.

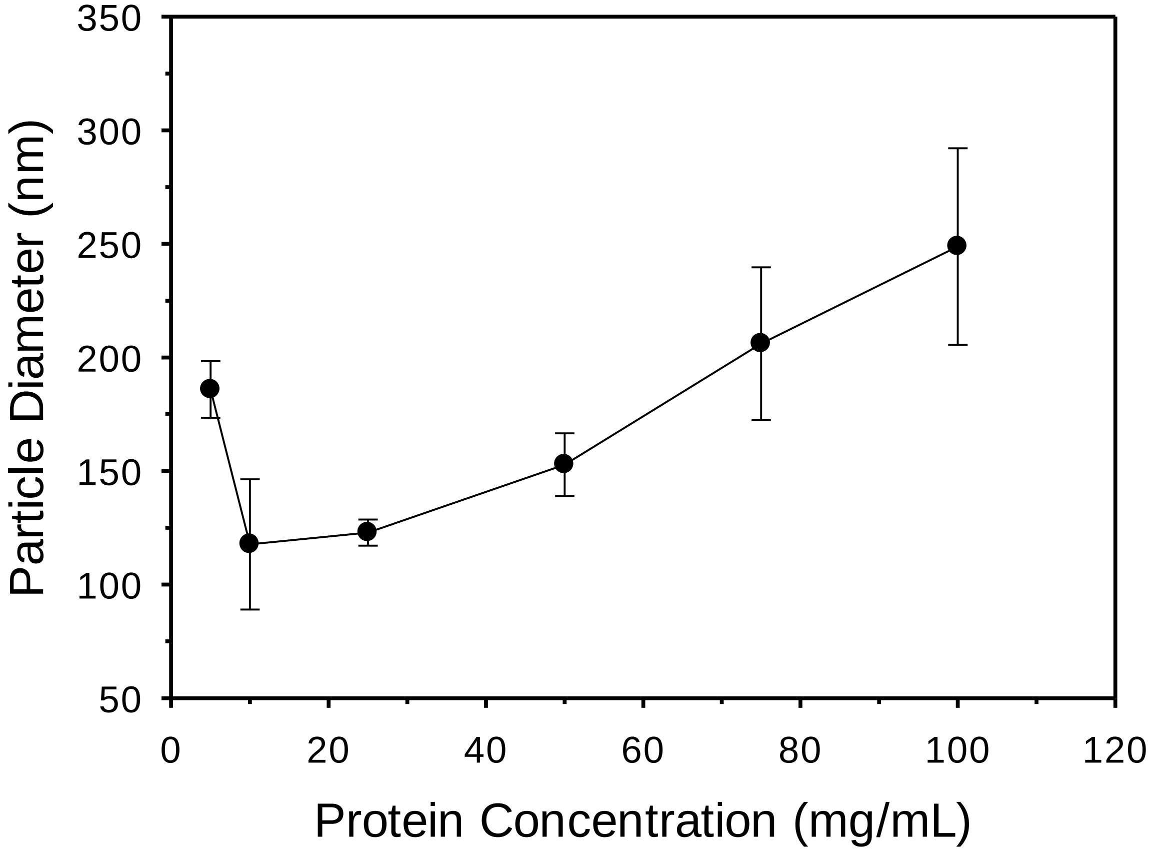

Supplemental Figure S2. Effect of initial dodecanoic acid-loaded bovine serum albumin (BSA-DF-C12) concentration on the mean nanoparticle diameters of cross-linked BSA nanoparticles measured with nanoparticle tracking analysis. Data represents the mean of three measurements from a single fabrication with error bars representing the standard deviation.

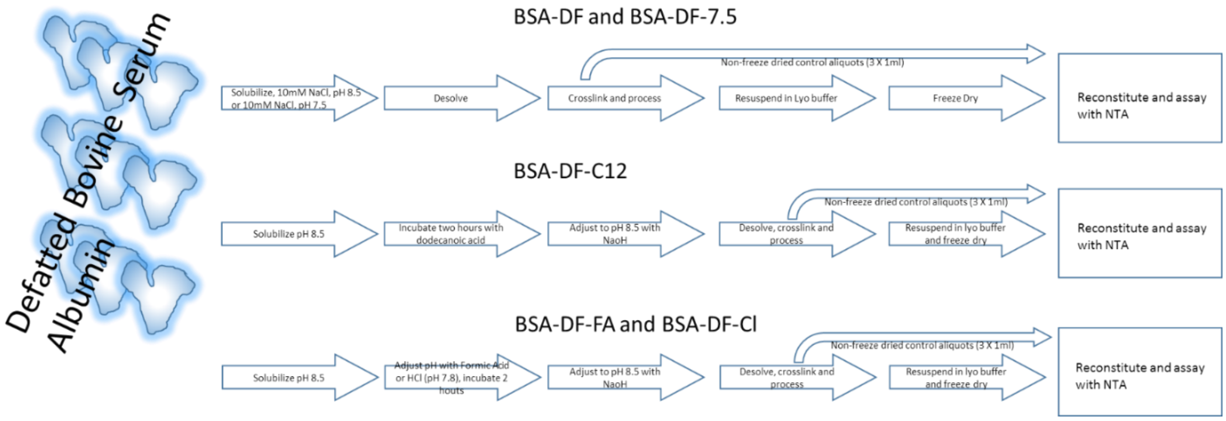

Supplemental Figure S3. Scheme for the production and assessment of Bovine Serum Albumin nanoparticle.
